# Exploring the Therapeutic Potential of Gamma-Aminobutyric Acid in Stress and Depressive Disorders through the Gut–Brain Axis

**DOI:** 10.3390/biomedicines11123128

**Published:** 2023-11-24

**Authors:** Timur Liwinski, Undine E. Lang, Annette B. Brühl, Else Schneider

**Affiliations:** University Psychiatric Clinics Basel, Clinic for Adults, University of Basel, CH-4002 Basel, Switzerland; timur.liwinski@upk.ch (T.L.); undine.lang@upk.ch (U.E.L.); annette.bruehl@upk.ch (A.B.B.)

**Keywords:** nutrition, mood disorder, neurotransmitter, microbiome, nutraceuticals, microbiome, gut–brain axis, enteric nervous system, insomnia, stress

## Abstract

Research conducted on individuals with depression reveals that major depressive disorders (MDDs) coincide with diminished levels of the inhibitory neurotransmitter γ-aminobutyric acid (GABA) in the brain, as well as modifications in the subunit composition of the primary receptors (GABA_A_ receptors) responsible for mediating GABAergic inhibition. Furthermore, there is substantial evidence supporting the significant role of GABA in regulating stress within the brain, which is a pivotal vulnerability factor in mood disorders. GABA is readily available and approved as a food supplement in many countries. Although there is substantial evidence indicating that orally ingested GABA may affect GABA receptors in peripheral tissues, there is comparatively less evidence supporting its direct action within the brain. Emerging evidence highlights that oral GABA intake may exert beneficial effects on the brain and psyche through the gut–brain axis. While GABA enjoys wide consumer acceptance in Eastern Asian markets, with many consumers reporting favorable effects on stress regulation, mood, and sleep, rigorous independent research is still largely lacking. Basic research, coupled with initial clinical findings, makes GABA an intriguing neuro-nutritional compound deserving of clinical studies in individuals with depression and other psychological problems.

## 1. Introduction

Depressive disorders are among the most common mental disorders; it is estimated that about 5% of adults worldwide suffer from depression [1]. During a depressive episode, a person experiences a depressed mood (feeling sad, irritable, empty) or a loss of pleasure or interest in activities for most of the day, nearly every day, for at least two weeks. Several other symptoms are also present, which may include poor concentration, feelings of excessive guilt or low self-worth, hopelessness about the future, thoughts about dying or suicide, disrupted sleep, changes in appetite or weight, and feeling especially tired or low in energy [2]. In addition to these symptoms, depression is characterized by cognitive impairment. For instance, deficits in episodic memory are associated with higher depression scores and pathological changes in depression [3,4]. Depression-related cognitive dysfunctions are generally associated with a higher rate of relapse [5], seen as a major factor contributing to functional impairments [6], and frequently persist after remission of a major depressive episode [7].

Globally, depression is a leading cause of disability and is thus a major contributor to the worldwide overall disease burden. The economic burden of major depressive disorder was estimated to amount to USD 326.2 billion in the United States alone [8]. Depression affects women more frequently than men. Depression can lead to suicide. A psychiatric diagnosis is present in about 98% of individuals committing suicide, with mood disorders being the leading cause with 30.2% [9]. Depression is often categorized into mild, moderate, and severe forms, for all of which effective treatments exist. However, many depressed individuals receive no mental health care, especially in low- and middle-income countries, where 75% of patients receive no treatment [10]. Most contemporary treatment guidelines recommend evidence-based psychological therapy as a first-line treatment for ‘milder’ presentations of depression and a combination of psychological and pharmacological therapy for moderate-to-severe depression [11,12,13]. Acknowledging the indispensable role of psychotherapy in depression, even in the so-called treatment refractory depression, this review focuses on treatment with psychotropic agents [14]. Antidepressants are first-line pharmacotherapy in most guidelines for the management of severe depression [11,12,13]. For patients who do not respond to the initial antidepressant prescribed, guidelines recommend alternative pharmacotherapeutic strategies (e.g., switching or augmenting with additional antidepressants and/or antipsychotic medications). Hence, despite increasing emphasis on psychological interventions supported by patients expressing a preference for these, in reality, antidepressant therapy is the mainstay of depression management, while psychotropic polypharmacy is the norm—especially in severe and complex mood disorders [15,16]. Despite substantial advances in treatment and management strategies for major depression, less than 50% of patients respond to first-line antidepressant treatment or psychotherapy [17]. If there is a lack of response to, or tolerability of, initial treatment, alternatives can be tried. In other instances, acute responses may be obtained, but subsequent relapses occur even on treatment [17,18]. “Treatment Resistant Depression (TRD)” is a term that has been used for several decades [19]. In the last couple of years, a new but related term has arisen: “Difficult-to-Treat Depression (DTD)” [20]. In such cases, neurostimulation techniques, especially electroconvulsive therapy (ECT), show a considerable success rate [21]. However, ECT carries the additional risk of anesthesia, its availability is still limited in many countries, and despite firm evidence for its effectiveness, it is not universally accepted by the public [21,22,23,24]. Given the high individual suffering and societal burden, there is a strong rationale for identifying novel preventive measures and treatment approaches to treat depression.

In this review, we aim to examine the neurobiological evidence supporting the role of the neurotransmitter gamma-aminobutyric acid (GABA) in depression, stress, sleep, and cognitive function. We also aim to summarize the evidence regarding the potential beneficial effects of oral GABA on stress and mood regulation, with the goal of exploring its potential as a neuro-nutraceutical for depression. Additionally, we will focus on GABA’s production by commensal and probiotic microorganisms and its interaction with the enteric nervous system, emphasizing its actions through the gut–brain axis.

## 2. GABAergic Hypothesis in Depression

Growing scientific findings suggest a correlation between major depressive disorders (MDDs) and a variety of GABAergic deficiencies. The GABAergic hypothesis of MDD proposes that changes in GABAergic neurotransmission are fundamentally significant elements in the development of depression’s underlying causes [25]. Major depression’s pathophysiology is still largely elusive, despite accumulating data highlighting alterations in various central nervous neurotransmitter systems. GABA is a non-proteinogenic amino acid and a firmly established inhibitory neurotransmitter abundant in the central nervous system of animals [26]. In recent years, GABA has become a hot topic in medical and pharmaceutical studies that highlight its multiple beneficial physiological effects, including neuroprotection, stress relief [27], promotion of sleep, neuronal cell injury prevention [28,29], antioxidant properties [30], blood pressure regulation [31,32], and protection from cancer [33]. GABA is produced from the excitatory neurotransmitter glutamate (Glu) in the brain [34]. GABAergic neurons are present throughout all levels of the neuraxis, representing between 20 and 40% of all neurons depending on the brain region and are known to balance and fine-tune excitatory neurotransmission of various neuronal systems [25]. GABA is the predominant inhibitory neurotransmitter in the mature brain [35]. GABAergic neurons account for one-third of the total synapses in the CNS, crucially impacting neural network dynamics [36]. GABAergic neurons play pivotal roles in key processes altered in psychiatric disorders, such as neural plasticity, sensory processing, stress reactivity, memory, and attention [36,37]. In contrast to Glu, which triggers the depolarization of postsynaptic neurons inducing an excitatory postsynaptic potential, GABA induces the hyperpolarization of postsynaptic neurons, resulting in an inhibitory postsynaptic potential [34]. GABA exerts its effects by activating two entirely different classes of receptors: the ionotropic GABA_A_ receptors (GABA_A_Rs) and the metabotropic GABA_B_Rs. GABA_A_Rs are known as key control elements of the anxiety state based on the potent anxiolytic activity of benzodiazepines [38,39]. Alterations of the GABAergic system are involved in numerous psychiatric disorders, including major depressive disorder, schizophrenia, bipolar affective disorder, and autism spectrum disorder [25,40,41,42].

Clinical studies with depressed individuals using magnetic resonance spectroscopy (MRS) and positron emission tomography (PET) have identified alterations in Glu and GABA concentrations and activity, suggesting that dysfunction in excitatory and/or inhibitory neurotransmitter signaling mechanisms may play a critical role in depression [43,44]. Using proton MRS (^1^H-MRS), an in vivo imaging technique for total tissue detection of neurochemicals, including N-acetylaspartate, GABA, Glu, glutamine (Gln), and a combination of Glu/Gln with a minor contribution from GABA (known as Glx), aberrant amino acid neurotransmitter levels measured by ^1^H-MRS have been found in patients with major depression [45,46,47,48]. Compared to healthy subjects, these differences have become especially apparent in the dorsolateral prefrontal cortex (PFC) [49]. In a meta-analysis of 17 ^1^H-MRS studies investigating patients with major depression, reductions of Glx in the PFC were associated with a number of failed antidepressant treatments, a measure of chronicity, and a proxy for severity of depressive illness course [48]. This finding is complemented by the detection of a selective loss of calbindin-positive GABAergic interneurons in dorsal PFC inpatients with depression [50]. Moreover, the GABAergic deficit hypothesis is supported by the detection of reduced plasma GABA levels [51,52], as well as a GABA reduction in cerebrospinal fluid and resected cortical tissue samples from depressed individuals [53,54]. Interestingly, GABA deficits seem to be most pronounced in treatment-resistant cases [55]. While altered Glu levels now show synchronicity with current mood, GABA levels seem to correlate with mood and show similar levels between remitted patients with major depression and subjects without a history of depression [56]. A pivotal role of glutamatergic/GABAergic neurotransmission in the pathophysiology of and treatment response to major depression is supported by strong evidence for the antidepressant efficacy of ketamine, a N-methyl-D-aspartate (NMDA) receptor antagonist [57,58,59]. The most effective method to manage treatment-resistant/difficult-to-treat depression is electroconvulsive therapy (ECT), although ECT’s precise mechanism of action is unclear. Evidence suggests that ECT’s strong antidepressant effect might be mediated by an increase in GABAergic activity [60]. GABA antagonists such as pregabalin showed efficacy against major depression and depressive symptoms as an adjunct treatment [61,62]. Other depression treatment modalities, such as cognitive behavioral therapy and selective serotonin reuptake inhibitors, also seem to restore the GABAergic deficit in major depression [63,64]. Many studies have reported that the inhibition of GABA signals allows for the continuous release of corticotropin-releasing factor (CRF) by paraventricular nucleus (PVN) neurons, resulting in cortisol overexpression and HPA axis hyperactivity, thus suggesting that such GABAergic activity is a key neurological factor in the maintenance of non-negative, anti-depressive emotional states [65].

## 3. GABA and Cognitive Function in Depression

Low GABA levels are also considered to play a crucial role in the cognitive symptoms of depression. The inhibitory system of GABAergic interneurons allocated throughout the hippocampus tightly controls and synchronizes hippocampal activity [66]. This modulation of hippocampal activity is a key mechanism for controlling neuronal plasticity and the ability to learn [67,68]. Specifically, somatostatin (SST) expressing GABAergic interneurons and alpha 5 GABA_A_ receptors, which are highly present in the hippocampus [69], play a crucial role in cognitive function [70]. Reduced SST expression, for instance, is associated with impaired cognition in normal aging [71] and Alzheimer’s disease [72]. It is believed that the low expression of SST reduces the excitatory signal-to-noise ratio and, thus, the synchronization of cellular and neural activity in the hippocampus, resulting in cognitive dysfunctions [73,74]. Augmenting SST+ cell post-synaptic α5-GABA-A receptor activity, on the other hand, ameliorates stress- and age-related cognitive dysfunction by balancing the hippocampal inhibitory/exhibitory activity [75,76]. Interestingly, SST-expressing neurons seem to be typically affected in MDD-related GABAergic phenotypes [77,78]. Therefore, low GABA levels are considered one of the most promising endophenotypes for therapeutic targets in depression [78].

## 4. GABA and the Microbiota–Gut–Brain Axis

The microbiome–gut–brain axis (MGBA) describes the bidirectional communication between the gastrointestinal tract, including its resident microbiota and the brain, linking emotional and cognitive centers of the brain with peripheral intestinal functions. The human gut microbiome consists of trillions of symbiotic bacteria that play a key role in regulating the host brain and behavior [79]. The precise mechanisms underlying this bidirectional regulation between the gut microbiome and the brain are still open to discussion, but the vagus nerve, the endocrine and immune system, and the synthesis and metabolism of metabolites and neurotransmitters in the gut are critically involved (reviewed in [80]). Moreover, merging evidence suggests that the gut microbiome plays a causal role in the etiology and psychopathology of MDD [81,82]. Two independent studies claimed that rodents who received fecal microbiome transplantation (FMT) from depressed patients had a higher level of inflammation and acted in a more depressive-like manner than rodents who received FMT from healthy volunteers [83,84]. Further FMT studies in rats also underscore the causal role of the gut microbiota in cognition. FMT from old to young rats impaired cognition and reduced brain-derived neurotrophic factor (BDNF) expression [85], whereas FMT from young rats to old rats ameliorated cognition and affected the hippocampal metabolome [86]. Numerous taxonomic alterations in bacterial composition have been identified in individuals diagnosed with MDD and in animal models designed to simulate depressive states [87]. These alterations include species-specific microbial shifts, such as increased levels of *Eggerthella*, *Paraprevotella*, *Flavonifractor*, and *Holdemania*, and decreased levels of *Christensenellaceae_R-7_group*, *Coprococcus*, *Fusicatenibacter*, and *Lachnospiraceae_ND3007_group* in humans. In rodent models, there is a reproducible reduction in *Acetatifactor*. Additionally, a disrupted balance of the microbiome and functional changes are consistently observed across species. This disruption is characterized by an increase in pro-inflammatory bacteria, like *Desulfovibrio* and *Escherichia/Shigella*, and a decrease in anti-inflammatory bacteria that produce butyrate, such as *Bifidobacterium* and *Faecalibacterium*. At the species taxonomic level, the abundance of *Bacteroides fragilis*, *Eggerthella lenta*, and *Ruminococcus gnavus* was found to be elevated only in patients suffering from depression. In depressed mice, there was an increased presence of *Mucispirillum schaedleri* and *Helicobacter rodentium* [87].

Generally, the gut microbiome can be modulated by diet and nutritional supplements, like live bacteria (probiotics) and non-digestible food components (prebiotics) [88,89]. There is evidence that regular intake of a probiotic supplement improves depressive symptoms [90,91]. The probiotic supplements most frequently consist of bacteria belonging to the genera *Lactobacillus* and *Bifidobacterium* [90,92], followed by genera such as *Lactococcus*, *Enterococcus*, *Streptococcus*, and *Leuconostoc* [91,93]. Interestingly, all of these bacterial genera are classified as lactic acid bacteria, which are mainly used for the fermentation of raw food [94]. Thus, the consumption of fermented food seems to have beneficial effects on mood [95]. The group of prebiotics, probiotics, and postbiotics (metabolites produced by the microbiome) that, when ingested, confer mental health benefits through interactions with commensal gut bacteria are defined as psychobiotics [96]. GABA, a crucial ‘postbiotic’ found in the enteric nervous system, is extensively distributed in foods and beverages and contributes significantly to MGBA functions and related disorders, including depression, anxiety, inflammatory, and cardiovascular disorders [93]. Prior research has demonstrated that bacteria have the capability to synthesize gamma-aminobutyric acid (GABA), a significant inhibitory neurotransmitter in the brain, through two distinct mechanisms. On one hand, a series of enzymes can convert arginine, ornithine, and agmatine into putrescine, and subsequently into GABA. This process involves GABA serving as an intermediate in the production of succinate via the GABA shunt pathway. This mechanism represents a means for bacteria to utilize carbon and nitrogen sources when faced with limited nutrient availability [97]. On the other hand, GABA synthesis can occur through the glutamate decarboxylase (GAD) system. In this pathway, a key enzyme, which is dependent on pyridoxal-5′-phosphate and encoded by gadA or gadB, converts glutamate into GABA. This conversion results in the production of carbon dioxide (CO_2_) and the consumption of protons [98,99]. In an initial survey of the Integrated Microbial Genomes/Human Microbiome Project database, it was revealed that there are 26 distinct bacterial genera that contain orthologs of the gadB gene. Notably, this includes Bacteroides, which is known to be one of the most abundant and widespread genera in the gastrointestinal tract (GIT) [99]. Strandwitz et al. subsequently validated this discovery using a collection of 1159 gut bacterial genomes representing diverse taxonomic groups [100]. Within this dataset, they identified 45 strains of Bacteroides that possessed orthologs of the gadB gene. Notably, GABA production was experimentally confirmed in six of these strains [100]. Through a combination of 16S rRNA sequencing and functional magnetic resonance imaging (fMRI) in individuals diagnosed with MDD, the authors observed an inverse relationship between the relative abundance of Bacteroides in the fecal microbiota and patterns in the brain that are linked to depressive symptoms [100].

**Figure 1:**
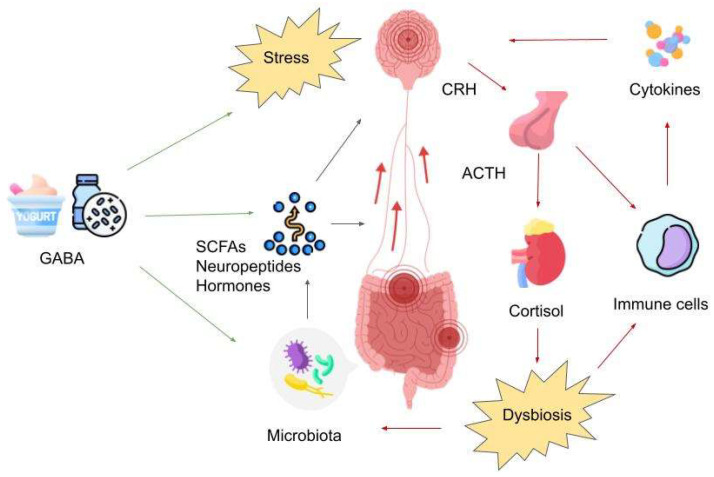
The diagram illustrates the intricate relationship between the depressive gut–brain axis and the impact of GABA on this axis. Prolonged exposure to chronic stress activates the hypothalamic–pituitary–adrenal (HPA) axis, resulting in the production of cortisol. This hormonal response disrupts the equilibrium of the gut microbiota and subsequently leads to increased permeability of the intestinal lining, commonly referred to as the “leaky gut.” Consequently, potentially harmful substances, notably lipopolysaccharides (LPS), gain access to the brain. Moreover, cortisol not only triggers an inflammatory reaction but also activates the endocannabinoid system. Concurrently, the disturbed gut microbiota generates various bioactive compounds, including neuropeptides, hormones, and short-chain fatty acids (SCFAs). The effects of these substances, in conjunction with those of inflammatory mediators, are predominantly mediated through the vagus nerve. The stress-induced activation of the HPA axis contributes to dysbiosis and heightened intestinal permeability, exacerbating the “leaky gut” condition. Consequently, proinflammatory cytokines further stimulate the HPA axis by influencing vagal nuclei (NTS/DMN) while also modulating the tryptophan-kynurenine pathway and altering neurotransmitter metabolism. It is noteworthy that GABA is not only consumed but also synthesized directly by specific genera of gut bacteria, particularly *Bifidobacterium* and *Lactobacillus*. Therefore, the administration of GABA, such as through fortified foods or probiotic bacteria capable of producing GABA, has the potential to restore the equilibrium of the gut microbiome. This restoration may facilitate the normalization of gut–brain communication via the enteric nervous system and vagus nerve, ultimately alleviating stress-related effects in the brain and improving depressive symptoms. Abbreviations: GABA: Gamma-Aminobutyric Acid; CRH: Corticotropin-Releasing Hormone; ACTH: Adrenocorticotropic Hormone; SCFAs: Short-Chain Fatty Acids.

Pathways of the MGBA, such as the vagus nerve, prevertebral sympathetic ganglia, and the hypothalamic–pituitary–adrenal axis, might be important mechanisms affected by GABA (see Figure 1). For instance, GABA-producing Lactobacilli have been found to reduce anxiety behaviors and markers of depression in mice and alter the expression of GABA_A_ receptor subunits in key brain regions that are involved in regulating mood and anxiety, such as the hippocampus and amygdala. The study also demonstrated the importance of vagal afferents in communicating GABAergic activity from the gut to the brain, as these modulatory effects were prevented in vagotomized mice [101,102,103].

## 5. GABA’s Impact on the Enteric Nervous System

The reciprocal communication between the brain and the Enteric Nervous System (ENS) plays a crucial role in preserving homeostasis [103]. Commensal bacteria belonging to *Lactobacillus* and *Bifidobacterium* strains have the potential to elevate GABA levels within the ENS [104]. GABA and its receptors are broadly distributed throughout the ENS [105]. Significant communication occurs between the gut and the brain via the vagal nerve [106]. An experiment conducted in mice revealed that the introduction of *Lactobacillus rhamnosus* (JB-1) consistently influenced the mRNA expression of GABA_Aα2_, GABA_Aα1_, and GABA_B1b_ receptor subunits [101]. These receptors are frequently linked to anxiety-like behavior [107]. The application of these bacteria resulted in a decrease in stress-induced corticosterone levels compared to the control group. Importantly, none of these effects were observed in mice that had undergone vagotomy [101]. In humans, the primary application of stimulating the vagus nerve through vagus nerve stimulation (VNS) has been in the treatment of refractory epilepsy [108]. VNS has obtained FDA approval for depression treatment. The realm of VNS is expanding, and rapid advancements in noninvasive VNS are noteworthy [109]. VNS is likely to influence multiple neurotransmitter systems in the brain [108]. GABA_A_ receptors could potentially play a role in the therapeutic effectiveness of VNS. In a study employing single photon emission computed tomography (SPECT) using the benzodiazepine receptor inverse agonist iomazenil to assess cortical GABA_A_ receptor density (GRD), researchers examined 10 individuals with drug-resistant partial epilepsy before and one year after the implantation of a VNS device. The findings indicated a significant correlation between therapeutic responses to VNS and the restoration of GRD to normal levels [110]. VNS also appears to elevate the concentration of unbound GABA in the cerebrospinal fluid [111].

These data suggest that while GABA may not directly cross the blood–brain barrier in humans, an indirect influence through the ENS could potentially provide a viable pathway for the impact of GABA dietary supplements. Although the connection between oral GABA administration, the vagal nerve, and GABA levels in the brain has not been firmly established, considering the existing evidence, it represents a promising avenue for future research.

## 6. Traditional Diets and Their Impact on Mood

The processes required for the production of fermented foods are ancient [112]. Modern humans’ paleolithic ancestors had plenty of access to food items subject to natural microbial fermentation (such as honey, fruits, berries, and their juices) long before a biochemical understanding of fermentation has emerged. Long before the discovery of microbes and the underlying biochemical processes, it has been realized that fermentation renders certain foods palatable, analgesic, and psychotropic or ensures their preservation [113]. Since the dawn of culture, fermented foods and beverages have become valuable cultural goods aiding human nutrition, traditional medicine, and certain ritual practices [114,115,116]. There is plenty of evidence suggesting that besides alcohol production, the fermentation of cereals, dairy, vegetables, fish, and meats were an integral element of ancestral diets [117]. Despite the introduction of chemical preservatives and refrigeration, fermented foods still account for up to one-third of consumed foods globally [118]. Mounting evidence highlights that fermentation can enhance the nutritional value of a wide variety of foods [119]. As knowledge of the human gut microbiome grows rapidly, the crucial impact that fermented food items exert on commensal microbes, with important health implications, is uncovered inch by inch.

Many authors conceive depression to be a “disease of modernity” [120], highlighting the notion that a shift away from traditional lifestyles is responsible for its rising prevalence [121,122]. This theory is related to the so-called evolutionary mismatch hypothesis [123], which assumes that the rising incidence of diseases such as depression arises from a flagrant discrepancy between the environments in which humans have evolved and adapted anatomically, and the modern environment. Food is among the paramount factors defining modern environments and is markedly different from pre-modern conditions [124]. The research community has shown increasing interest in food as a variable influencing mood and mental health [122]. It seems obvious that, given the brain’s dependence on nutrition to provide for its structure and energy metabolism, diet is expected to be paramount for mental health. Thus, one would expect human nutrition to be a major focus of mental health research. In reality, however, there is a dearth of research, particularly high-quality research, on the role of diet in mental health. The so-called field of nutritional psychiatry has historically been neglected and is populated with mostly poorly designed studies [119]. For millennia, diverse plant and animal products were subjected to fermentation by various bacteria, yeasts, and fungi to produce palatable foods [125]. As such, fermented products are an essential part of many traditional diets. When discussing the health implications of a traditional diet, the Japanese and Mediterranean models receive considerable interest [126,127,128,129,130,131,132]. The traditional Japanese diet is rich in various fermentation products, such as foods containing probiotic bacteria, black rice vinegar (kurosu), soy sauce (shoyu), soybean-barley paste (miso), natto, and tempeh. These foods are produced by traditional methods that harness mixed cultures of various microorganisms such as lactic acid bacteria, acetic acid bacter sake yeast, koji molds, and natto bacteria [133]. Several population studies have established a link between adherence to traditional dietary practices and a lower risk of anxiety and depression [134,135,136,137,138,139]. Adherence to a traditional Japanese dietary custom has been linked with lower depressive symptom rates [130,131]. A substantial body of evidence indicates that the Mediterranean diet is protective against depressive symptoms and major depression [140,141]. This finding is supported by population surveys and randomized controlled trials (RCTs) [142,143,144,145,146,147,148]. Mediterranean diet’s health benefits might be explained by its high content of antioxidants, fibers, monosaturated and omega-3 fatty acids, phytosterols, and probiotic microorganisms [149,150]. However, the Mediterranean diet is also rich in lacto-fermented foods (that is, foods fermented by lactic acid bacteria), such as lacto-fermented pickles, and dairy products, such as yoghurt and cheese [151]. The health benefits of lacto-fermented foods have received considerable attention, and they might be a crucial factor underlying the Mediterranean diets’ health-promoting effects [151]. Additional foods characteristic of the Mediterranean diet that contain GABA include items such as beans, tomatoes, spinach, mushrooms, and buckwheat [152].

## 7. Fermented Foods Enriched with GABA

Many fermented foods are rich in GABA. Lactic acid bacteria and yeasts exploited in fermented food production show GABA-yielding properties [153]. Numerous GABA-producing microorganisms have been identified in fermented products. For example, *Lactobacillus brevis* J1 is a lactic acid bacterial strain isolated from fermented cow milk; it accumulates up to 9.87 g/L GABA when cultured in Man, Rogosa, Sharpe (MRS) medium [154]. *Lactobacillus plantarum* M-6 strain that was isolated from traditional Chinese fermented food displays good GABA-producing traits by accumulating 545.33 mg/L GABA while inoculated in MRS medium with chickpea milk, fortified with monosodium glutamate [29]. Other than *Lactobacillus* spp., *Bacillus cereus* strain KBC was also isolated from fermented soy moromi, shown to produce a maximum GABA value of 532.74 mg/L in MRS broth for 7 days of fermentation [155]. The Streptococcus thermophilus APC151 strain accumulates accumulate 2.1 mg/mL GABA and is suitable for the manufacture of GABA-enriched bioactive yogurt [156].

Regarding probiotics, *Lactobacillus rhamnosus* strains are among the best characterized probiotic microorganisms. *L*. *rhamnosus* is a rod-shaped, facultative heterofermentative and anaerobic commensal bacterium abundant in the gastrointestinal tract. It is believed to contribute to the maintenance of gut homeostasis [157]. *L*. *rhamnosus* is well known for its GABA-producing properties [158]. Strains of *L*. *rhamnosus*, such as JB-1 and HN001, can regulate depressive states in both humans and mice; particularly, the JB-1 strain has been shown to increase cortical GABAergic activity in mice [101,159,160]. *L*. *rhamnosus* (JB-1) increases CNS GABA levels in mice, modulating GABA_A_ and GABA_B_ receptor expression by activating GABA signaling pathways via vagal afferents [100,161]. *L*. *rhamnosus* GG has been found to increase GABA concentration within fermented adzuki bean milk under optimized cultural conditions [162]. In a study investigating the beneficial properties of quinoa yogurt beverages, *Lactobacillus rhamnosus* SP1 and *Lactobacillus plantarum* T6B10 raised the beverages’ GABA levels up to 211 mg/kg [163]. An RCT evaluated the effect of *Lactobacillus rhamnosus* HN001 (HN001) given in pregnancy and postpartum on symptoms of maternal depression and anxiety in the postpartum period. Women who were administered HN001 exhibited notably reduced depression and anxiety scores during the postpartum period [160].

## 8. GABA-Enriched Fermented Foods as Neuro-Therapeutics

The growing global demand for functional (probiotic) dairy foods can be largely attributed to the high level of interest consumers have in food products that promote health. Yogurt has gained widespread consumer acceptance and is widely regarded as the ideal medium for delivering beneficial functional ingredients [164,165], whereas GABA has emerged as a promising bioactive ingredient in functional foods [166]. Due to its numerous physiological functions and positive effects on metabolic disorders, GABA has been the subject of substantial research [167]. One of its most significant benefits is its demonstrated hypotensive effect in animal studies and human intervention trials [168]. The biosynthesis of GABA and its optimization, while preserving sensory characteristics, are crucial factors in creating GABA-enriched food products that offer health benefits. Lactic acid bacteria (LAB) are the primary producers of GABA, making it possible to create a wide range of GABA-enriched fermented foods that are natural, safe, and eco-friendly. The increased understanding of bioactive compounds in food has opened up new opportunities for the development of naturally occurring functional foods that offer added health benefits [153]. Due to its lipophilic and charged nature at physiological pH, GABA is unable to easily cross the blood–brain barrier passively [169]. This has led to a debate over whether oral administration of GABA can directly affect brain function, with some studies suggesting that the actions of oral GABA may be attributed to its effects on the enteric nervous system [93]. Earlier studies found that, under normal circumstances, intravenous, intraperitoneal, or oral administration of GABA did not increase brain GABA levels [170,171]. However, recent studies in mice have identified a GABA transporter, GAT2/BGT-1, responsible for GABA transport across the blood–brain barrier, and further research is needed to explore GABA blood–brain barrier permeability in humans [172]. Despite this, there is extensive evidence that oral supplementation with GABA can reach the brain and exert biological effects in both humans and animals, including mood improvement and activity in the central nervous system. High plasma concentrations of GABA have also been shown to increase GABA concentrations in the brain [173]. For instance, in rats, one month of oral GABA supplementation enhanced novel object recognition memory and working memory [174]. Similarly, in healthy human adults at rest, the administration of 100 mg GABA in water increased the alpha:beta wave ratio measured by EEG, indicating improved relaxation [27]. Additionally, oral GABA administration during a mental stress task resulted in a smaller decrease in alpha waves, suggesting an acute stress-reducing effect in human adults [175]. In another study, GABA-enriched yeast supplementation altered the balance between cortical excitation and inhibition, as shown by EEG in humans [176]. There are various possible mechanisms through which oral GABA may exert its effects. GABA_A_ receptors can be modulated by a range of steroids, including neuroactive steroids and neurosteroids, which are synthesized in the brain [177]. As such, exogenous GABA may indirectly affect GABA activity in the brain by acting on these steroids and other modulators [178]. Moreover, GABA may act as a source of energy via the GABA shunt, which can bypass the usual TCA (tricarboxylic acid) cycle and increase ATP (adenosine triphosphate) [179].

The human body is capable of producing its own GABA. However, factors such as a deficiency in estrogen, zinc, or vitamins, as well as an excess of salicylic acid and food additives, can inhibit the body’s ability to produce GABA [180]. GABA-enriched food is required since the GABA content of daily diets is rather low [181]. A GABA concentration as low as 2.01 mg in 200 mL of GABA-enriched Oolong tea showed stress decreasing effects in high-stressed individuals [178]. Consumption of 100 mg of biosynthetic GABA for a week might improve sleep value in human subjects with poor sleep quality [182]. A dosage of 300 mg biosynthetic GABA from fermented rice germ helps to shorten sleep latency [183]. Abdou and colleagues investigated the potential of orally administered GABA to promote relaxation and immunity during times of stress [27]. Two studies were conducted, the first of which evaluated the effect of GABA intake on the brain waves of 13 subjects. Each volunteer underwent 3 tests—one with only water, one with GABA, and one with L-theanine. Electroencephalograms (EEG) were taken after each test, and the results showed that after 60 min, GABA significantly increased alpha waves and decreased beta waves when compared to water or L-theanine. This indicates that GABA not only induces relaxation but also reduces anxiety. The second study examined the role of GABA intake as a relaxant and anxiolytic agent in promoting immunity in stressed volunteers. Eight acrophobic subjects were divided into two groups, one receiving a placebo and the other GABA. Both groups were required to cross a suspended bridge as a stressful stimulus, and their saliva was monitored for levels of immunoglobulin A (IgA) during the crossing. The placebo group exhibited a marked decrease in IgA levels, while the GABA group showed significantly higher levels. In conclusion, GABA has demonstrated potential as a natural relaxant, with effects that can be observed within an hour of administration, and as a means of reducing anxiety. Furthermore, GABA administration may enhance immunity under conditions of stress [27]. Kanehira and colleagues studied the effects of a GABA-rich beverage on occupational fatigue [184]. The authors assigned an arithmetic task for the Uchida-Kraepelin Psychodiagnostic Test (UKT) to 30 healthy Japanese subjects, 9 of whom were diagnosed as having chronic fatigue. The subjects were administered 250 mL of a test beverage containing GABA at doses of 0, 25, and 50 mg before assigning tasks for the UKT. Psychological fatigue assessed by the Visual Analogue Scale (VAS) was significantly lower in the group administrated the beverage containing 50 mg GABA than in the control group. The results of the Profile of Mood States (POMS) also indicated that psychological fatigue was significantly reduced in the 50-mg GABA group. The salivary secretion levels of chromogranin A and cortisol—markers of physical fatigue—in both the 25-mg and 50-mg GABA groups were significantly lower than those in the control group. The 50-mg GABA group also showed a higher score on UKT by solving the arithmetic task more accurately than the control group. The results suggest that the intake of GABA-containing beverages, especially those containing 50 mg of GABA, may help reduce both psychological and physical fatigue and improve task-solving ability [184]. In a 4-week RCT employing biosynthetic GABA, an elevation in cortisol levels was observed in the placebo group at both the 2nd and 4th weeks of GABA use. Conversely, the group receiving GABA did not exhibit such an increase, emphasizing the stress-reducing properties of GABA [185]. Yamatsu et al. investigated the impact of GABA added to coffee on stress and fatigue through a randomized, double-blind, placebo-controlled, crossover-designed study. Nineteen participants consumed coffee and coffee enriched with 28 mg of GABA as test samples, engaging in a 17-min arithmetic task to induce stress and fatigue. Salivary chromogranin A (CgA) was measured to assess stress levels before and after the arithmetic task. GABA added to coffee demonstrated stress-reducing and fatigue-relieving effects [186].

Nakamura et al. assessed both CgA and HRV and found that 28 mg of GABA in 10 g of chocolate, compared to 20 g of chocolate alone, decreased LF/HF power 6.5–9.5 min after the arithmetic task and increased HF power 12–15 min after the task [187]. In post-menopausal women, the consumption of 26.4 mg of GABA rice three times a day, compared to control rice, improved insomnia scores in the Kupperman Menopause Index after 4 weeks of treatment [188]. Byun et al. conducted a prospective, randomized, double-blind, and placebo-controlled trial with 40 patients experiencing insomnia symptoms. After 4 weeks of treatment, the GABA group exhibited decreased sleep latency and increased sleep efficacy compared to the placebo group. Side effects have rarely been reported in pharmaceutical-grade GABA studies [183]. Byun et al. noted mild abdominal discomfort (*n* = 2), headache (*n* = 1), and drowsiness (*n* = 1) in 3 out of 40 GABA-treated patients [183].

Moreover, GABA-enriched foods are considered to improve memory and learning abilities [189]. A particularly promising functional food is fermented *Laminaria japonica* (FLJ), a sea tangle typically used as a food resource in Pacific and Asian regions because of its high contents of dietary fiber, carbohydrates, minerals, and protein. Using a specific LAB fermentation process involving *Lactobacillus brevis* BJ20, the glutamic acid contained in the sea tangle is bio-converted into GABA [190]. There is convincing evidence that the consumption of FLJ has beneficial effects on cognition. An intake of 1 g/day of FLJ containing 5% GABA for eight weeks significantly increased the brain-derived neurotrophic factor (BDNF), a biomarker tightly linked to hippocampal neurogenesis and memory, in elderly women compared to a placebo group [190,191,192,193]. If FLJ (5% GABA) was consumed for 6 weeks (1.5 g/day), global cognition, working memory, logical reasoning, information processing, and selective attention also improved significantly in elderly women compared to a placebo group. In mice, the supplementation of FLJ ameliorated short-term memory impairment and the hippocampal-dependent spatial learning ability to an equivalent degree as conventional therapy in scopolamine-, ethanol-, and trimethyltin chloride-induced dementia [190,194]. Moreover, there is evidence that *kefir*, a traditional fermented milk beverage from the Caucasus mountains produced by adding a kefir grain to milk, increases the gut microbiota’s capacity to produce GABA in mice. On the behavioral side, the consumption of kefir improved reward learning and fear-dependent contextual memory [195]. Even though all these studies reported convincing evidence that GABA-enriched foods can improve cognition, there are also studies reporting null or even negative results. Tınok et al. could not find a positive effect of a GABA supplement (800 mg) dissolved in orange juice on spatial attention and visual working memory [196]. In addition, Leonte et al. did not find a positive effect of GABA supplement (800 mg) on spatial attention but on temporal attention [197]. However, the improvement in temporal attention could not be replicated by Tınok et al. [196].

Interestingly, there are no studies investigating the beneficial effect of GABA-enriched foods on cognitive deficits in depression. Nevertheless, two studies examined the effect of probiotic supplements on cognitive symptoms in depressed patients [198,199]. Both studies found improved verbal episodic memory after consumption of a probiotic supplement for either four or eight weeks. In both studies, the administered supplements contained *Lactobacillus* and *Bifidobacterium* strains, such as *Lactobacillus plantarum* and *Bifidobacterium brevi*, which are known to produce GABA [93]. Thus, it may well be assumed that an increased production of GABA was responsible for the improvement in hippocampal-dependent verbal episodic memory. However, there is currently a lack of direct evidence to confirm this.

## 9. Conclusions

It is possible that the quantity of GABA reaching the brain may be too minimal to have clinical relevance but sufficient to produce an effect in a stop-change paradigm. Nonetheless, oral GABA obtained from oral supplements, probiotics, GABA-rich fermented foods, or fortified food products may exert an effect on the brain through complex peripheral mechanisms, primarily involving the Enteric Nervous System (ENS) and the gut–brain axis. We maintain the belief that Magnetic Resonance Spectroscopy (MRS) studies offer the most auspicious approach for directly evaluating the impact of GABA supplementation on GABA levels in the human brain.

Notably, in one of the rat studies under consideration, the administration of GABA alone led to a 33% increase in brain GABA levels. However, when GABA was administered in conjunction with L-arginine, brain GABA levels surged by an impressive 383.3% [200]. It would indeed be intriguing to investigate whether this effect can be replicated in humans, potentially serving as a leverage point to enhance the efficacy of GABA in human applications. Considering the widespread distribution of GABA and its receptors in peripheral tissues, it is highly justified to conduct further research into the impact of oral GABA on peripheral tissues and the gut–brain axis as part of the investigation into the potential benefits of GABA-enriched foods.

The dietary supplement form of GABA is readily accessible to consumers. While many individuals assert that they derive advantages from using these products, it remains uncertain whether these supplements provide benefits beyond what could be attributed to a placebo effect. There is some supportive evidence suggesting a calming impact of GABA dietary supplements; however, it is worth noting that much of this evidence comes from researchers who may have a potential conflict of interest [169]. Furthermore, it is important to mention that in some of the studies identified, the sample sizes were relatively small, which can limit the strength of the conclusions that can be drawn [187]. Additional evidence from independent studies is required to conclusively establish the favorable psychological effects of GABA. LD50 tests conducted on rats using a 5000 mg/kg dose of natural GABA did not result in any fatalities [201]. This demonstrates a favorable safety profile, rendering it an appealing adjunct therapeutic option deserving of further comprehensive study. However, it is evident that additional safety data covering various dosage ranges in humans are clearly needed. Our research reveals a dearth of evidence regarding the efficacy of pharmaceutical-grade GABA in addressing depression. Nevertheless, RCTs investigating the impact of GABA on stress, sleep, and cognition prompt exploration in the context of depression, given the frequent impairment of these domains in individuals experiencing depressive states. Unfortunately, the credibility of numerous scrutinized RCTs is compromised by potential conflicts of interest, small participant cohorts, and imbalances in the control and intervention groups. Moreover, the heterogeneity in the assessment of stress and sleep parameters, as well as variations in study designs, precludes the conduct of a quantitative meta-analysis due to the disparate nature of the extracted data. Lastly, the limited and diverse nature of studies in this realm precludes the establishment of an optimal dose for efficacy in stress and sleep benefits, both within the scope of this review and in the broader scientific literature. Consequently, our review advocates future investigations to delve into dose-response relationships between oral consumption of natural and biosynthetic GABA and targeted parameters such as stress, sleep, cognitive performance, and mood. This exploration should employ diverse methodologies, including self-reporting, behavioral assessments, peripheral measurements, and neurophysiological markers of stress and sleep.

In summary, the various potential physiological benefits of GABA in mood regulation, stress tolerance, and cognitive performance, coupled with its favorable safety profile, make GABA an intriguing natural compound worthy of investigation in prospective clinical trials for depression.
